# Role of L‐type Ca^2+^‐channels in the vasorelaxing response to finerenone in arteries of human visceral adipose tissue

**DOI:** 10.14814/phy2.70062

**Published:** 2024-09-24

**Authors:** Francesca Schinzari, Alessandro De Stefano, Giuseppe Sica, Marco Mettimano, Carmine Cardillo, Manfredi Tesauro

**Affiliations:** ^1^ Department of Aging Fondazione Policlinico Universitario Agostino Gemelli IRCCS Rome Italy; ^2^ Department of Systems Medicine Tor Vergata University Rome Italy; ^3^ Department of Experimental Medicine Tor Vergata University Rome Italy; ^4^ Department of Cardiovascular Sciences Fondazione Policlinico Universitario Agostino Gemelli IRCCS Rome Italy; ^5^ Department of Translational Medicine and Surgery Catholic University Rome Italy; ^6^ Department of Neurosciences Fondazione Policlinico Universitario Agostino Gemelli IRCCS Rome Italy

**Keywords:** adipose tissue, calcium channels, finerenone, mineralocorticoid receptor, obesity

## Abstract

Inadequate blood supply to the expanding adipose tissue (AT) is involved in the unhealthy AT remodeling and cardiometabolic consequences of obesity. Because of the pathophysiological role of upregulated mineralocorticoid receptor (MR) signaling in the complications of obesity, this study tested the vasoactive properties of finerenone, a nonsteroidal MR antagonist, in arteries of human AT. Arteries isolated from the visceral AT of obese subjects were studied in a wire myograph. Finerenone resulted in a concentration‐dependent relaxation of arteries precontracted with either the thromboxane‐A2 analog U46619, ET‐1, or high‐K^+^ solution; the steroidal MR antagonist potassium canrenoate, by contrast, did not relax arteries contracted with either U46619 or high‐K^+^ solution. Finerenone‐induced relaxation after precontraction with U46619 was greater in the arteries of obese versus nonobese subjects. Mechanistically, the vasorelaxing response to finerenone was not influenced by preincubation with the nitric oxide synthase inhibitor L‐NAME or by endothelium removal. Interestingly, finerenone, like the dihydropyridine Ca^2+^‐channel blocker nifedipine, relaxed arteries contracted with the L‐type Ca^2+^‐channel agonist Bay K8644. In conclusion, finerenone relaxes arteries of human visceral AT, likely through antagonism of L‐type Ca^2+^ channels. This finding identifies a novel mechanism by which finerenone may improve AT perfusion, hence protecting against the cardiometabolic complications of obesity.

## Graphical abstract

The vasoactive response to the nonsteroidal mineralocorticoid receptor antagonist (MRA) finerenone was tested ex vivo in arteries of human visceral adipose tissue (VAT). Finerenone elicited endothelium‐independent vasorelaxation in arteries precontracted with different vasoconstrictors; the steroidal MRA potassium canrenoate, by contrast, did not relax those arteries. Both finerenone and the L‐type calcium channels (LTCCs) antagonist nifedipine relaxed arteries contracted with the LTCCs agonist BAY K8644. These findings suggest that blockade of LTTCs, rather than antagonism of the mineralocorticoid receptor, is the likely mechanism of the finerenone‐induced relaxation in human VAT arteries.

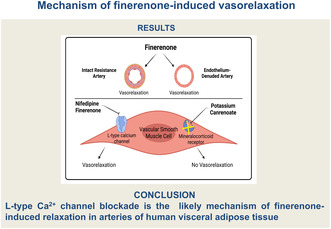

## 1 INTRODUCTION

Obesity is associated with increased activity of the systemic and tissue renin‐angiotensin‐aldosterone system (RAAS), which plays an important role in the development of insulin resistance, hypertension, type 2 diabetes (T2D), and cardiovascular disease (Jia et al., 2017). In particular, obesity‐related hyperproduction of aldosterone within adipose tissue (AT), through activation of the mineralocorticoid receptor (MR), may promote adipogenesis and the creation of a hypoxic milieu (Briones et al., 2012; Dinh Cat et al., 2016). This maladaptive AT expansion is associated, in turn, with higher oxidative stress, due to increased reactive oxygen species and reduced antioxidant capacity (Furukawa et al., 2004; Lefranc et al., 2018), thereby contributing to insulin resistance (Pellegrinelli et al., 2016). Moreover, stimulation of MR may promote AT inflammation via increased macrophage recruitment and higher production of pro‐inflammatory cytokines (Dinh Cat et al., 2016; Kargi & Iacobellis, 2014). In addition, soluble aldosterone‐releasing factors secreted by adipocytes may lead to systemic overproduction of aldosterone (Faulkner et al., 2018), which participates in obesity‐related vascular dysfunction by augmenting the vasoconstrictor tone, through reduced bioavailability of nitric oxide (NO) and increased expression of endothelin‐1 (Schmidt et al., 2003). Taken together, these data emphasize the therapeutic potential of MR targeting in obesity, a view strengthened by the observations that treatment with steroidal MR antagonists (MRAs) decreases obesity‐associated vascular inflammation, oxidative stress, and arterial stiffening (DeMarco et al., 2015).

Despite their favorable actions on AT remodeling and, more in general, on cardiovascular morbidity and mortality (Ivanes et al., 2012), clinical use of steroidal MRAs is limited by their hormonal side effects and the risk of hyperkalemia. In this regard, the discovery of nonsteroidal MRAs has represented a major therapeutic advance. Thus, these drugs differ from their steroidal counterpart for important pharmacodynamic and pharmacokinetic characteristics (Agarwal et al., 2021), including higher selectivity and potency of MR antagonism (Savarese et al., 2024), while maintaining beneficial anti‐inflammatory, anti‐remodeling, and anti‐fibrotic properties in the kidney, the heart, and the vasculature (Kintscher et al., 2022). Importantly, recent, large‐scale, clinical trials have demonstrated that the nonsteroidal MRA finerenone improves chronic kidney disease (CKD) outcomes in patients with type 2 diabetes (T2D) (Bakris et al., 2020) and reduces cardiovascular events in patients with CKD and T2D (Pitt et al., 2021).

The present study was designed to assess the vasoactive effects of the nonsteroidal MRA finerenone in resistance arteries isolated from visceral adipose tissue (VAT) of either obese or nonobese humans and to investigate the mechanisms underlying the vascular action of finerenone.

## 2 METHODS

### 2.1 Chemicals and solutions

U46619 (Tocris Bioscience, Cat# 1932, Bristol, United Kingdom), finerenone (MedChemExpress, Cat# HY‐111372, Monmouth Junction, NJ, USA), BayK8684 (Tocris Bioscience, Cat# 1544), and nifedipine (Tocris Bioscience, Cat# 1075) were dissolved in dimethyl sulfoxide. Bradykinin (BK; Sigma‐Aldrich, Cat# B3259, St. Louis, MO, USA), L‐N^G^‐nitroarginine methyl ester (L‐NAME, Cat# N5751, Sigma‐Aldrich), ET‐1 (Calbiochem, Cat# 05‐23‐3800, St. Louis, MO, USA), and potassium canrenoate (Neopharmed Gentili, Milano, Italy) were dissolved in distilled H_2_O. Physiological salt solution (PSS) contained (mmol L^−1^): NaCl 115; NaHCO_3_ 25; K_2_HPO_4_ 2.5; MgSO_4_ 1.2; glucose 5.5; HEPES 10; and CaCl_2_ 1.3 (pH 7.4). High‐K^+^ PSS was obtained as a mix of PSS and a solution containing NaCl 20 mmol L^−1^ and KCl 95 mmol L^−1^, to reach, as desired, a K^+^ concentration of 32 mmol L^−1^ or 8 mmol L^−1^ in the organ chamber. Buffers were continuously aerated with 5% CO_2_ in air at 37°C. All the indicated concentrations of the pharmacological agents added to the bathing solution represent their final concentration in the organ chamber.

### 2.2 Participants

Visceral AT (VAT) biopsies were collected from 22 individuals (10 males, 12 females) undergoing laparoscopic surgery (cholecystectomy, hernial repair, or metabolic surgery); 16 of these subjects were obese (body mass index [BMI] 40.8 ± 1.2 Kg/m^2^), six were nonobese (BMI 25.4 ± 1.2 Kg/m^2^; *p* < 0.001). Systemic blood pressure and the biochemical parameters assessed on blood samples collected in a fasting state before surgery (plasma glucose, fibrinogen, renal function tests, and liver enzymes) were not different between the two groups (all *p* > 0.05).

### 2.3 Adipose tissue collection and vessel preparation

VAT biopsies were placed in ice‐cold PSS, and either immediately processed or stored overnight in 30 mL PPS at 4°C. Arteries were isolated from the tissue in PSS at room temperature and cleaned of surrounding fat and connective tissue, according to a procedure reported elsewhere (Bloksgaard et al., 2015). The obtained vessels (lumen diameter ranging from 317 to 722 μm, average 527 ± 30 μm) were then cut into 2–4 segments of ≈2 mm length and mounted into the chambers of a wire myograph (DMT, Aarhus, Denmark) containing 5 mL of PSS at 37°C. Afterward, arteries were kept for 15–30 min at 37°C to perform the force calibration, with the arterial segments stretched to obtain a diameter and a wall tension corresponding to a transmural pressure of 100 mmHg. The internal diameter of the arteries was calculated, the viability of the vessels was tested by adding the high‐K^+^ solution, and the presence of intact endothelium was assessed by testing relaxation to BK (1 μmol L^−1^). Segments that failed to contract or relax were discarded. The Lab Chart Pro software (AD Instruments, Dunedin, New Zeeland) was used to record and analyze the vasomotor responses.

### 2.4 Evaluation of the vascular responses to finerenone in arteries of obese subjects contracted with different vasoconstrictor agents

Arteries prepared as described above, after a washout period to re‐establish the baseline tone, were contracted using either the stable synthetic analog of the thromboxane A2 U46619 (1 μmol L^−1^; *n* = 13), ET‐1 (3 nmol L^−1^; *n* = 6) or a high‐K^+^ solution (32 mmol L^−1^; *n* = 5); after 5–10 min, changes in the vascular tone in response to increasing concentrations of finerenone (10^−7^–10^−4^ mol L^−1^) were determined.

### 2.5 Comparison of vascular response to finerenone between arteries of nonobese and obese individuals

To evaluate whether obesity affects the vascular responses to finerenone, after preparation, arteries obtained from six nonobese and 13 obese individuals were contracted with U46619 and, after 5–10 min, cumulative concentration‐response curves (CCRCs) to finerenone (10^−7^–10^−4^ mol L^−1^) were obtained in the two groups.

### 2.6 Assessment of the vascular effect of the steroidal MR antagonist potassium canrenoate

To assess whether antagonism of MR may play a role in the vasorelaxation elicited by finerenone, arteries obtained from VAT of obese individuals were contracted with either U46619 (1 μmol L^−1^; *n* = 3) or a high‐K^+^ solution (32 mmol L^−1^; n = 3); after 5–10 min, the CCRC to the steroidal MR antagonist potassium canrenoate (10^−7^–10^−4^ mol L^−1^) was determined.

### 2.7 Possible involvement of endothelium in the vasorelaxing response to finerenone

To investigate the possible role of endothelium‐derived NO in the vascular response to finerenone, its effect was assessed by pre‐incubating arteries of obese subjects for 15 min with the nitric oxide (NO) synthase inhibitor L‐NAME (100 μmol L^−1^), before constricting them with either U46619 (1 μmol L^−1^; *n* = 4) or ET‐1 (3 nmol L^−1^; *n* = 3) and then constructing a CCRC. To determine the potential involvement of other endothelium‐derived relaxing substances, arteries (*n* = 3) of obese subjects were denuded of endothelium by surface abrasion, gently inserting and removing a nylon suture (Ethilon 8–0; Ethicon, Somerville, NJ, USA) for 50× in the lumen, before mounting them in the myograph chamber; the success of the endothelial removal procedure was confirmed by absent relaxation of vessels to 1 μmol L^−1^ BK following precontraction with U46619 (1 μmol L^−1^); thereafter, arteries were contracted with U46619 (1 μmol L^−1^) and the CCRCs to finerenone (10^−7^–10^−4^ mol L^−1^) were compared between segments with intact or denuded endothelium.

### 2.8 Characterization of the mechanisms of finerenone‐induced vasorelaxation in VAT arteries

In order to gain more insights into the mechanisms involved in the vasorelaxing effect of finerenone observed in VAT arteries, given some similarities of its molecular structure with that of the dihydropyridine Ca^2+^‐channel blockers (CCBs) (Yang & Young, 2016), experiments were performed to assess whether it might act through blockade of L‐type (voltage‐operated) Ca^2+^‐channels (LTCCs). To this end, in arteries of obese subjects contracted with either U46619 (1 μmol L^−1^), the CCRC to the dihydropyridine CCB nifedipine (10^−7^–10^−4^ mmol; *n* = 4) was compared with that of equimolar doses of finerenone. In addition, after incubation with potassium chloride (8 mmol L^−1^) for 10 minutes to obtain mild depolarization and a more stable contraction (Piascik & Babich, 1986), arteries of obese subjects were contracted with the dihydropyridine Ca^2+^‐channel agonist BAY K8644 (1 μmol L^−1^) and CCRCs to either finerenone (*n* = 4), nifedipine (*n* = 3), or potassium canrenoate (*n* = 3), at the same concentrations as before, were obtained.

### 2.9 Histology

To rule out the possibility that finerenone might exert indirect anti‐contractile actions by antagonizing vasoconstricting substances released from perivascular adipose tissue (PVAT) (Hu et al., 2021), segments of a sample vessel were formalin‐fixed and stained with hematoxylin–eosin for histological analysis, to confirm that no residual PVAT had been left in place after the procedure used before mounting the vessels in the wire myograph.

### 2.10 Immunohistochemistry

Samples (*n* = 3) were formalin‐fixed and embedded in paraffin. Serial sections were used for the immunohistochemistry staining to study the expression of MRs. Paraffin sections (3 μm thick) were treated with citrate buffer pH 7.8 for 30 min at 95°C for antigen retrieval. Afterward, sections were incubated with an anti‐MR receptor mouse monoclonal antibody (Abcam, Cat# AB2774, Cambridge, UK) for 60 min. Reactions were detected using a HRP‐DAB Detection Kit (UCS Diagnostic, Cat# HSL60‐DAB, Roma, Italy). An Axioscope 5 light microscope (Zeiss, Oberkochen, Germany) was used to evaluate the immunohistochemical reactions as the number of MR‐positive cells. Reactions were set up using specific positive and negative control tissues (in particular, kidney tissue and visceral adipose tissue were used as positive controls and prostate tissue as a negative control).

### 2.11 Statistical analysis

For the CCRC experiments, differences between the dose‐tension relations were analyzed using 2‐way ANOVA or 2‐way ANOVA for repeated measures, followed by the Holm‐Sidak post‐hoc test for multiple comparisons, as appropriate. Changes in the dose‐tension relations from baseline were analyzed by one‐way ANOVA for repeated measures. Other comparisons were tested by Student's *t*‐test. The potency of inhibition (EC_50_) of Bay K8644‐induced contraction by the CCRCs to finerenone or nifedipine was calculated by non‐linear regression analysis. A *p* value <0.05 was considered statistically significant. CCRCs are reported as percent of relaxation. Statistical analyses were performed by use of the SigmaPlot software (SPSS Inc., Chicago, IL, USA). As commonly done in vascular reactivity studies (Briones et al., 2012; Michea et al., 2005; Mueller et al., 2015), data are reported as means ± standard error of the mean (SEM).

## 3 RESULTS

The data supporting the findings of this study are available from the corresponding author upon reasonable request.

### 3.1 Vascular response to finerenone in arteries pre‐contracted with different vasoconstrictor agents

**FIGURE 1:**
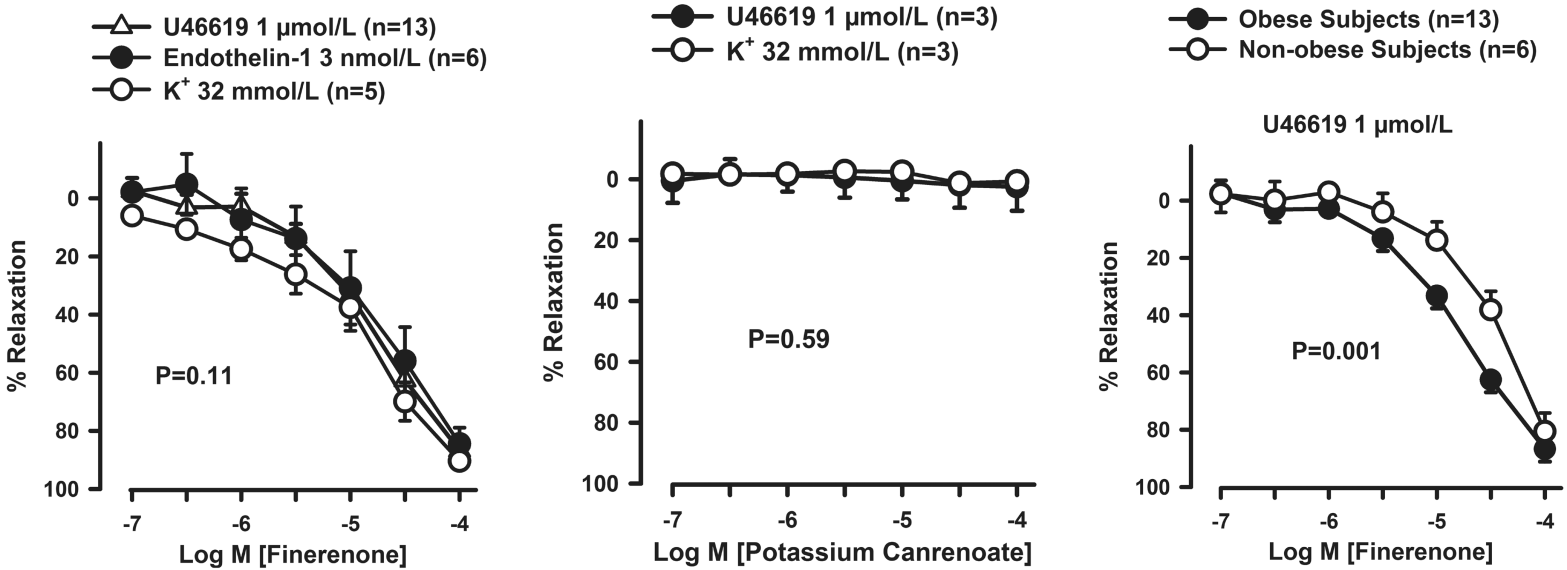
The left panel depicts the cumulative concentration‐response curves to finerenone in arteries of obese subjects precontracted with U46619, ET‐1, or high‐K^+^ physiological salt solution. The middle panel depicts the cumulative concentration‐response curves to potassium canrenoate in arteries of obese subjects precontracted with either U46619 or high‐K^+^ physiological salt solution. The right panel compares the cumulative concentration‐response curves to finerenone between arteries from nonobese and obese patients. Data are reported as means±SEM. The *p* value indicates the significant differences in the dose‐tension relations between contracting agents (left and middle panels) or between groups (right panel) at the 2‐way ANOVA.

Finerenone (10^−7^–10^−4^ mol L^−1^) resulted in a concentration‐dependent relaxation (all *p* < 0.001 vs. baseline) in arteries contracted with either U46619 (1 μmol L^−1^), ET‐1 (3 nmol L^−1^), or high‐K^+^ solution (32 μmol L^−1^), without significant difference among vessels contracted with the different agents (Figure 1, left panel).

### 3.2 Vascular action of the steroidal MR antagonist potassium canrenoate

In contrast with the finerenone results, increasing concentrations of the steroidal MR antagonist potassium canrenoate did not significantly relax arteries contracted with either U46619 or high‐K^+^ solution, without difference between the different agents (Figure 1, middle panel).

### 3.3 Comparison of the vasorelaxing responses to finerenone in arteries of nonobese and obese individuals

Finerenone induced a concentration‐dependent relaxation in arteries from visceral AT of both nonobese or obese individuals contracted with U46619; the relaxation induced by finerenone was significantly higher in the arteries of the obese than in those of control individuals (Figure 1, right panel).

### 3.4 Effects of NO synthase inhibition or endothelium removal on the vasorelaxing response to finerenone

**FIGURE 2:**
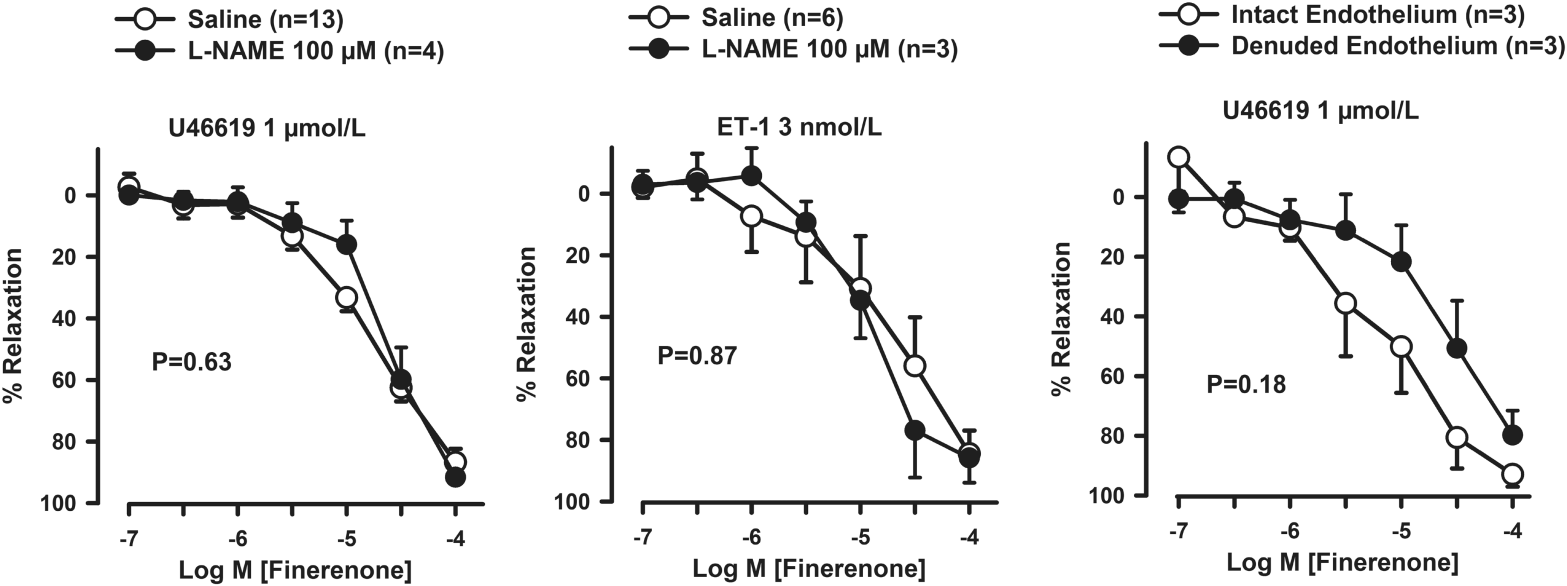
The left panel depicts the vasorelaxing response to finerenone in arteries of obese subjects contracted with U46619 (left panel) or high‐K^+^ physiological salt solution (middle panel), following preincubation with either saline or the nitric oxide synthase inhibitor L‐N^G^‐Nitroarginine methyl ester (L‐NAME). The right panel represents the comparison between the vasorelaxation induced by finerenone in arteries of obese patients with either intact or removed endothelium. Data are reported as means ± SEM. The *p* values indicate the significance level of differences in the dose‐tension relations at the 2‐way ANOVA (left and middle) or at the 2‐way ANOVA for repeated measures (right).

The vasorelaxing response to finerenone (10^−7^–10^−4^ mol L^−1^) was not significantly different in segments preincubated with L‐NAME (100 μmol L^−1^) and those preincubated with saline after contraction with either U46619 (10 μmol L^−1^; Figure 2, left panel) or ET‐1 (3 nmol L^−1^) (Figure 2, middle panel). As expected, bradykinin (BK, 1 μmol L^−1^)‐induced relaxation was almost completely abolished in segments of U46619‐contracted arteries with denuded, as compared with those with intact endothelium (*p* < 0.001 intact vs. denuded). The CCRC to finerenone, by contrast, was not different between segments with intact vs. denuded endothelium contracted with U46619 (Figure 2, right panel).

### 3.5 Involvement of L‐type Ca^2+^‐channels in the vasorelaxing effect of finerenone

**FIGURE 3:**
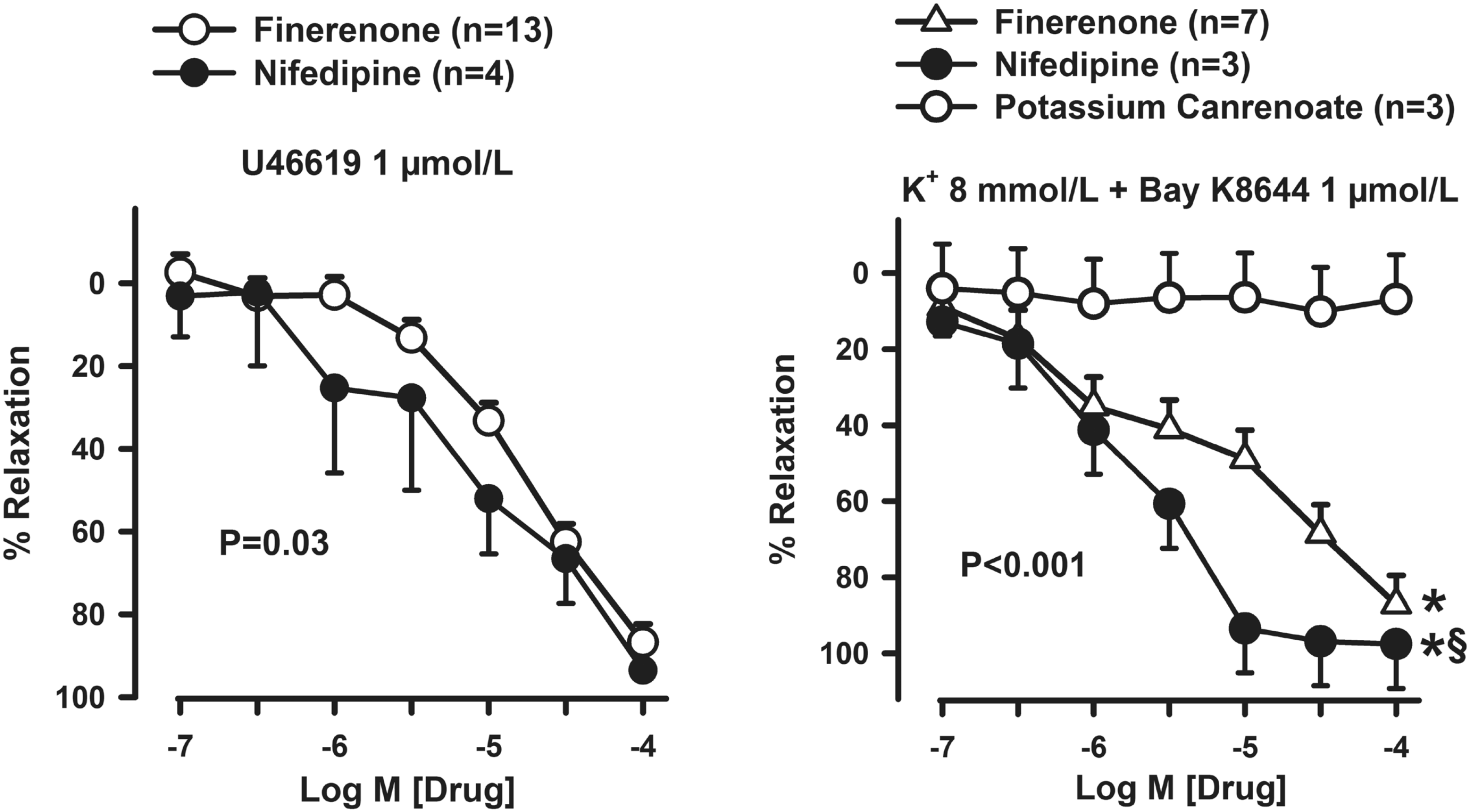
The left panel depicts the comparison of the vasorelaxing responses to finerenone and nifedipine in arteries of obese subjects contracted with U46619. The right panel compares the responses to finerenone, nifedipine, and potassium canrenoate in arteries of obese subjects contracted with the Ca^2+^‐channel agonist BAY K8644 after preincubation with 8 mmol L^−1^ of potassium chloride. Data are means±SEM. The *p* values indicate the significance level of differences at the 2‐way ANOVA. *p < 0.05 versus potassium canrenoate and ^§^ *p* < 0.05 versus finerenone at the post hoc analysis by Holm‐Sidak test for multiple comparisons.

In VAT arteries contracted with U44619, the dihydropyridine Ca^2+^‐channel blocker nifedipine resulted in a concentration‐dependent vasorelaxation (*p* < 0.001 vs. baseline). The relaxation induced by nifedipine in those vessels was slightly higher than that determined by equimolar concentrations of finerenone (Figure 3, left panel). Arteries pretreated with K^+^ 8 mmol L^−1^ for 10 min and then exposed to the LTCC Bay K8644 exhibited a stable contraction; when CCRCs to either finerenone or nifedipine (both from 10^−7^ to 10^−4^ mmol L^−1^) were constructed in those vessels, a dose‐dependent vasorelaxation was observed (both *p* < 0.001 vs. baseline), with a response significantly higher after nifedipine than finerenone; similarly, comparison between the two substances of the reduction on BAY K8644‐developed tone demonstrated significantly lower EC50 value of nifedipine than finerenone (2.6 ± 1.3 and 37.7 ± 19.9 μmol L^−1^, respectively; *p* < 0001). When those vessels, by contrast, were challenged with potassium canrenoate, no significant relaxation was observed (Figure 3, right panel).

### 3.6 Histology

Staining a sample vessel with hematoxylin–eosin confirmed that the procedure had left no residual PVAT before mounting the arterial segments in the myograph (not shown).

### 3.7 Immunohistochemistry

**FIGURE 4:**
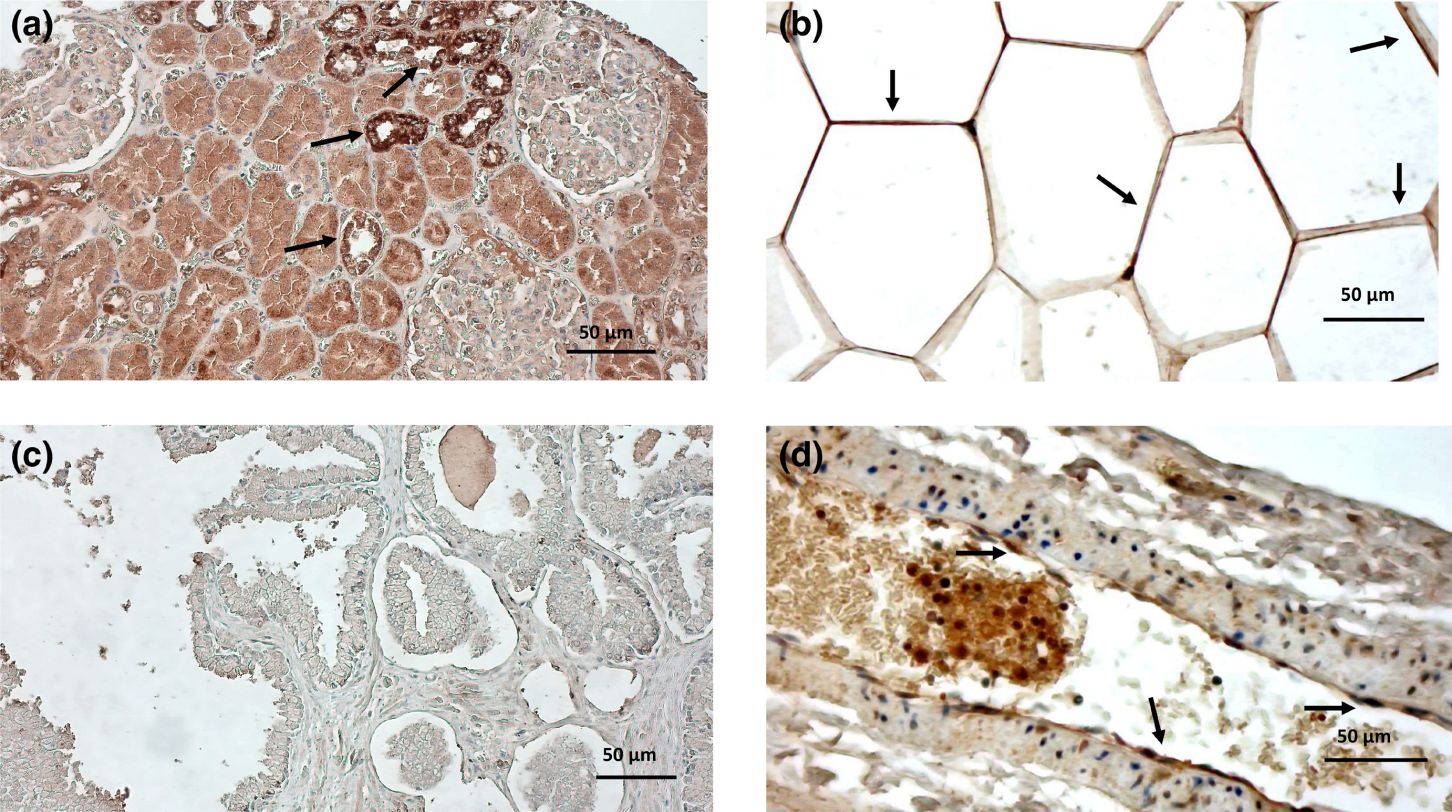
Immunohistochemistry shows the expression of MR (arrows) in epithelial cells of the renal tubule (a) and visceral AT adipocytes (b), but not in the prostate (c). In visceral adipose tissue resistance arteries (d), positive immunostaining for MR was detected in endothelial (arrows), but not in smooth muscle cells.

Positive staining for MR was found in epithelial cells of the renal tubule (Figure 4a) and in perivascular adipose tissue (Figure 4b), but not in prostatic tissue (Figure 4c). Analysis of intact vessels did not demonstrate any MR staining in vascular smooth muscle cells, while showing positive staining in endothelial cells (Figure 4d).

## 4 DISCUSSION

The present study demonstrates that finerenone relaxes resistance arteries of human VAT contracted with vasoconstrictor agents acting through disparate mechanisms, without difference in its effect on vessels contracted with either the thromboxane A2 analog U46619, ET‐1, or high‐K^+^ solution. The steroidal MRA potassium canrenoate, by contrast, was unable to relax arteries contracted with either U46619 or high‐K^+^ solution. Taken together, these results suggest that the mechanism underlying the vasorelaxing action of the nonsteroidal MRA finerenone is not shared by the steroidal MRA potassium canrenoate.

In our study, finerenone‐induced vasorelaxation was similar in the absence or presence of the NO synthase inhibitor L‐NAME, which allows us to rule out endothelium‐derived NO as the mediator of the vascular action of finerenone in VAT arteries. In addition, relaxation to finerenone was preserved in arterial segments where endothelium had been removed, thereby making unlikely the involvement of endothelium‐derived vasodilators different than NO. These observations clearly suggest that direct action on vascular smooth muscle cells (VSMCs) is the likely mechanism of finerenone‐induced relaxation in arteries of human VAT, even though the low number of vascular samples used in these experiments and their derivation exclusively from obese individuals reduce the emphasis of our results. As intracellular Ca^2+^ influx in VSMCs is a main mechanism in vasoconstriction (Brozovich et al., 2016), it is reasonable to postulate that the finerenone‐induced vasorelaxation observed in our study might relate to the inhibition of this phenomenon. Among different Ca^2+^ channels located in the plasma membrane of VSMCs (Touyz et al., 2018), LTCCs have been shown to constitute the main route of Ca^2+^ entry for control of myogenic tone and contraction (Ghosh et al., 2017). Interestingly, similar to other nonsteroidal MRAs, the molecule of finerenone includes the 1,4‐dihydropyridine ring characteristic of LTCC blockers, such as nifedipine (Perez‐Gordillo et al., 2023). In addition, it has been previously shown that several dihydropyridine CCBs are also provided of some MRA activity (Dietz et al., 2008). To test the assumption that finerenone may induce vasorelaxation by blockade of LTCCs, we compared the vascular responses to finerenone and nifedipine in arterial segments precontracted with the LTCC agonist BAY K8644. We observed that finerenone shares with nifedipine the ability to relax those vessels, thereby supporting the notion of LTCC blockade as the plausible mechanism underlying finerenone‐elicited vasorelaxation in human VAT arteries. The lower potency of finerenone, compared to nifedipine, in relaxing arteries contracted with BAY K8644, as seen in our study, might be explained by the fact that the 1,4‐dihydropyridine compounds with good MRA properties have reduced CCB activity (Arhancet et al., 2010). The specificity of the effect of finerenone as inhibitor of LTCC agonist‐induced contraction is reinforced by our observation that potassium canrenoate, which does not possess LTCC‐blocking properties, did not relax the arterial segments contacted with BAY K8644. However, because we did not measure whether preincubation with finerenone might concurrently affect changes in arterial tension and VSMC intracellular Ca^2+^ content (Sanchez et al., 2018) induced by increasing concentrations of BAY K8644, this limitation mitigates the strength of our results.

Previous studies in both experimental models and humans have demonstrated that the vasoconstrictor response to aldosterone involves predominantly the activation of the MR (DuPont & Jaffe, 2017). Thus, activation of the MR by aldosterone may result in some rapid, nongenomic effects, in addition to several slow, genomic effects (Ong & Young, 2017), with rapid MR signaling occurring not only in the renal epithelial cells, where it is involved in the regulation of sodium handling, but also in VSMCs of small arteries, where it importantly contributes to the maintenance of basal tone and determination of contractile responses. In VSMCs of the mesenteric artery, MR has been shown to enhance the activity of the LTCC Ca_v_ 1.2, thereby leading to higher vasoconstrictor tone (McCurley & Jaffe, 2012). Also, aldosterone has been shown to elicit rapid vasoconstriction of rat mesenteric resistance vessels by increasing intracellular Ca^2+^, through nongenomic mechanisms involving protein kinase C and phosphatidylinositol 3‐kinase (Michea et al., 2005). In addition, stimulation of the MR in VSMCs has been shown to affect sodium handling by up‐regulation of the sodium‐hydrogen exchanger (NHE)‐1, leading to enhanced sodium influx and a vasoconstrictor response (Carreno et al., 2015). We could not evoke, however, the mechanisms about MR‐induced vasoconstriction reported by these earlier observations to explain the vasorelaxing response to finerenone seen by us in human VAT arteries. Similarly, the greater vasorelaxing response to finerenone that we observed in the arteries of obese versus nonobese individuals could not be attributable to the upregulation of MR signaling previously reported in vessels of obese animals (Briones et al., 2012). This is because the lack of vasodilator response to the steroidal antagonist of MR, potassium canrenoate, in human VAT arteries precontracted with various vasoconstrictors argues against the involvement of MR signaling in the effects of finerenone. This view is supported by the results of our immunohistochemistry analyses, showing absent immunostaining for MRs in smooth muscle cells of VAT arteries. By contrast, we found those receptors expressed in the epithelial cells of the renal tubule, white adipocytes, and the endothelium of VAT arteries. The reasons for the discrepancies between the current and previous investigations about the presence or absence of MR‐mediated vasoconstrictor signaling are not entirely clear, even though differences between species, vascular beds, and vessel size may constitute possible explanations (Mueller et al., 2015). As to the greater potency of vasodilator activity of finerenone seen by us in vessels of obese versus nonobese individuals, it might possibly relate to an upregulation of Ca^2+^ entry through LTCCs, as observed in VSMCs of obese animals (Sanchez et al., 2018), as a possible consequence of increased oxidative stress (Touyz et al., 2018). Because our study did not collect direct evidence in this regard, however, other explanations cannot be excluded.

## 5 CONCLUSIONS

In summary, our study indicates that finerenone relaxes VAT arteries through an endothelium‐independent mechanism, with greater potency in arteries of obese versus nonobese subjects. This effect does not seem to involve antagonism of the MR, but likely relates to blockade of LTCCs.

Previous studies have demonstrated that finerenone improves clinical outcomes and reduces mortality in patients with disease states characterized by increased systemic activity of the RAAS, such as heart failure or kidney disease associated with T2D (Chilton & Silva‐Cardoso, 2023). In the last decades, however, it has become apparent that activation of the RAAS may occur in several tissues outside the adrenal glands and locally exert relevant pathogenic roles. Particularly, increased production of different components of the RAAS, including aldosterone, has been observed in the VAT of obese individuals, where it contributes to hypoxia, inflammation, increased oxidative stress, and fibrosis (AlZaim et al., 2023). These changes, in turn, might drive the metabolic derangement and cardiovascular complications of obesity (Akoumianakis et al., 2017). In this context, the role of the AT vasculature has gained considerable mechanistic relevance, due to the observation that vascular dysfunction, in conjunction with vessel rarefaction, may promote the obesity‐related maladaptive AT remodeling that often precedes the development of cardiometabolic disease (AlZaim et al., 2023). Consequently, the relaxing effect of finerenone on nutritive arteries of VAT seen in our study suggests that the drug might increase VAT perfusion and thus prevent unhealthy AT remodeling. This action might then translate into benefits against obesity‐associated metabolic and cardiovascular disease. It must be considered, however, that the concentrations of finerenone used in our study to fully relax VAT resistance arteries ex vivo were higher than those achievable in vivo following the oral administration of the drug. The hypothesis of cardiometabolic prevention afforded by finerenone in human obesity, therefore, requires direct testing by specifically designed clinical trials.

## AUTHOR CONTRIBUTIONS

F.S. was involved in conceptualization and writing. A.DS. was involved in methodology, software and data curation. G.S. was involved in resources. M.M. was involved in data curation. C.C. was involved in conceptualization, validation, supervision and funding acquisition. M.T. was involved in supervision and funding acquisition.

## FUNDING INFORMATION

This work was supported by a Beyond the Borders grant from University of Tor Vergata to M. Tesauro and by Fondi d'Ateneo grants (Linea D.1) from Catholic University to C. Cardillo.

## CONFLICT OF INTEREST STATEMENT

None.

## ETHICS STATEMENT

The study protocol was approved by the Institutional Review Board of the University of Rome Tor Vergata (approval number 13/2016), and all the participants gave written informed consent before they participated in the study.
